# Hepatitis D virus and hepatitis B virus infection in Amerindian communities of the Amazonas state, Colombia

**DOI:** 10.1186/s12985-015-0402-5

**Published:** 2015-10-24

**Authors:** Diana di Filippo Villa, Fabian Cortes-Mancera, Edra Payares, Neyla Montes, Fernando de la Hoz, Maria Patricia Arbelaez, Gonzalo Correa, Maria-Cristina Navas

**Affiliations:** Grupo de Gastrohepatologia, Facultad de Medicina, Universidad de Antioquia, UdeA, Calle 70 No. 52-21, Medellin, Colombia; Grupo de Investigación e Innovación Biomedica, Instituto Tecnológico Metropolitano, Medellin, Colombia; Laboratorio Departamental de Salud Publica de Amazonas, Leticia, Colombia; Coordinacion de Salud Publica, Alcaldia de Puerto Nariño, Amazonas, Colombia; Instituto Nacional de Salud, Bogota, Colombia and Grupo de Epidemiología y Evaluación en Salud Pública, Universidad Nacional, Bogota, Colombia; Grupo de Epidemiologia, Facultad de Salud Pública, Universidad de Antioquia, Medellin, Colombia; Present Address: Facultad de Investigación Judicial, Forenses y Salud, Tecnologico de Antioquia, Medellín, Colombia

**Keywords:** Hepatitis D virus, Hepatitis B virus, Amazonas state, Amerindian population, Superinfection, Genotypes, South America

## Abstract

**Background:**

In Colombia, cases of Hepatitis D virus (HDV) infection have been officially described since 1985 mainly in Amerindian population from Sierra Nevada de Santa Marta (North Caribbean Coast), Uraba (North West), and Amazon (South East). The last official report of a clinical case of HDV infection in Colombia was registered in 2005.

**Objectives:**

The aims of this study were to identify cases of HDV and/or Hepatitis B virus (HBV) infection in asymptomatic Amerindians from Amazonas state, South East Colombia, and to describe the circulating viral genotypes in this population.

**Study design:**

The study population was recruited in 19 Amerindian communities in the Amazonas state. Individuals over 18 years old were screened by rapid test for Hepatitis B surface Antigen (HBsAg). Blood samples obtained from individuals positives for HBsAg in the rapid-test assay were analyzed for HBsAg, anti-HBc, anti-HDV IgM/IgG by ELISA. The detection of HBV DNA and HDV RNA was performed by PCR amplification. The viral genotype was determined by sequencing and phylogenetic analysis.

**Results:**

A total of 23/861 individuals were positive for HBsAg detection by rapid test. Serological and/or molecular markers of HDV infection were demonstrated in 43.5 % (10/23) of samples from Amerindians. The phylogenetic analysis demonstrated the exclusive circulation of HBV subgenotype F1b of and HDV 3 in this population.

**Conclusions:**

A high frequency of HBV/HDV infection was found in Amerindian population from Amazonas State, Colombia (43.5 %, 10/23). Nine cases were identified in a population of 861 asymptomatic Amerindian individuals; one symptomatic case (with diagnosis of end-stage hepatic disease) was also identified in the study. The circulation of HDV 3 and HBV subgenotype F1b suggests a constant flow of these viral genotypes as a result of the interaction of the Amerindian populations from Amazon basin. Further studies are necessary to confirm whether HBV subgenotype F1b is the prevalent in the population from South East region in Colombia.

## Background

HDV is a defective virus, since it requires the presence of the Hepatitis B virus (HBV) surface antigen (HBsAg) for assembly of infectious viral particles [1]. HDV contains a small RNA genome with a single open reading frame (ORF) that codes for two isoforms of the delta antigen, small delta antigen (SDAg) and large delta antigen (LDAg). Two major specific patterns of HDV infection have been described: co-infection and superinfection [2, 3].

In South America, predominantly in Amazon Basin, cases of severe hepatitis and liver failure have been usually associated with HDV/HBV superinfection [4–9]. HDV infection are described in Colombia mainly in regions that have been considered highly endemic for HBV infection including Sierra Nevada de Santa Marta (North Caribbean Coast), Magdalena (North Caribbean Coast), Uraba (North West), Choco (Pacific Coast) and Amazonas (South East) [10–13].

Two important studies carried out in 1991 showed a very high prevalence of HBV infection in habitants from Magdalena state and Amazonas States. The first study was conducted in 4 towns (Riofrio, Santa Rosalia, Varela and Julio Zawady towns) considering the cases of liver failure cases reported in Magdalena state in that period. The prevalence of HBsAg reported in the population of these towns ranged from 9 % to 18 %. Moreover, the antibodies anti-HDV were detected from 6 % to 63 % in these populations [11]. The second study was conducted in 404 Amerindian children and adolescents belonging to the Huitoto ethnic group in Amazonas state. The overall prevalence of HBsAg was 18 %. Cases of liver failure were also reported in this ethnic group [14].

The last official report of a clinical case of HDV infection in Colombia was notified in Guainia State (South East) in 2005 [15]. Since then, no new hepatitis D cases have been reported to the surveillance system of public health in Colombia (SIVIGILA).

The incidence of HBV infection in Amazonas state in 2010 was 7.5 times higher (27.8/100.000) than the average incidence of the country (3.7/100.000) [16]. In 2014, an average incidence of 4.34/100.000 was reported while an incidence of 25.2/100.000 was notified in Amazonas state [17]. In this state, the circulation in 2011 of HDV genotype 3 in Amerindian population was documented [18], even though the last official report of a clinical case of HDV infection in the country was in 2005 [15].

The aims of this study were to identify cases of HDV and/or HBV infection in asymptomatic individuals from Amerindian communities in the Amazonas state and to describe the circulating viral genotypes in this population.

## Results

A total of 861 asymptomatic Amerindian individuals and 1 symptomatic (with diagnosis of end-stage hepatic disease), older than 18 years were recruited in the Amazonas state, South East Colombia. On average 39,4 % (range 6,4 %–90 %) of the population from each community was included in the study (Table 1).

**Table 1:** Study population and individuals positive for HBsAg and HDV in 19 Amerindian communities of the Amazonas state, Colombia

Municipality	Amerindian community		^a^Total population	Study population	HBV positive (HBsAg)	HDV positive (anti-HDV and/or viral genome)
	Tipisca		91	50 (54.9 %)	0	
	Puerto Rico		92	75 (81.5 %)	2 (2,6 %)	
	Santarem		23	10 (43.5 %)	1 (10 %)	
	Doce de Octubre		107	24 (22.4 %)	1 (4,1 %)	
	San Juan de Atacuari		159	58 (36,5 %)	3 (5,1 %)	1
	Siete de agosto		155	38 (24,5 %)	1 (2,6 %)	
Puerto Narino	Naranjales		205	37 (18,0 %)	1 (2,7 %)	1
	Puerto Esperanza		209	63 (30,1 %)	3 (4,7 %)	
	Veinte de julio		129	17 (13,2 %)	0	
	Patrulleros		54	46 (85,2 %)	1 (2,1 %)	1
	Boyahuazu		95	43 (45,3 %)	1 (2,3 %)	1
	Valencia		42	23 (54,8 %)	0	
	Ticoya		159	29 (18,2 %)	0	
		Total	1520	513	14 (2,7 %)	4 (28,5 %)
	San Martin de Amacayacu		NA	48	0	
	Santa Sofia		NA	37	2 (2,3 %)	1
Leticia	Arara		1107	33 (6,4 %)	0	
	Nazareth		1016	85 (20,4 %)	3 (8,1 %)	2
		Total	2123	203	5 (2,4 %)	3(60 %)
	Puerto Nuevo		121	109 (90 %)	1 (0,9 %)	1
Tarapaca	Ventura		144	37 (25,7 %)	3 (8,1 %)	2
		Total	265	146	4 (2,7 %)	3 (75 %)
		Total	6559	862	23	10

^a^Population >18 years old, data obtained from the database of the Expanded Program on Immunization (EPI). NA: Not available

Twenty-three individuals from 862 (2.6 %) were positive for HBsAg by the rapid test; all serum samples were also positive for HBsAg and Anti-HBc by Elisa. The average age of the HBV positive individuals was 32 years (SD ± 11.2 years); the stratification by age showed that 60.8 % corresponded to individuals between 22 and 31 years old. The proportion of men (52.2 %) and women (47.8 %) was similar.

The frequency of HBV-positive cases was comparable among the communities of Puerto Nariño (2.7 %; 14/513), Leticia (2.4 %; 5/203) and Tarapaca (2.7 %, 4/146) (Table 1). The 43.5 % (10/23) of these individuals declared having hepatitis and/or cases of hepatitis in the family; 69.6 % (16/23) of the individuals positive for HBsAg had not been vaccinated for hepatitis B (Table 2).

**Table 2:** Socio-demographic and exposure variables of Amerindians positive for HBsAg

Variables of study	N = 23	Percent
Age group		
22-31 years	14	(60,8 %)
32-41 years	6	(26,1 %)
>41 years	3	(13 %)
Gender		
Female	11	(47,8 %)
Male	12	(52,2 %)
Ethnic group		
Tikuna	17	(73,9 %)
Cocama	2	(8,7 %)
Yagua	4	(17,4 %)
Municipality		
Puerto Narino	14	(60,8 %)
Leticia	5	(21,7 %)
Tarapaca	4	(17,4 %)
Dental extraction or healing		
No	8	(34,8 %)
Yes	15	(65,2 %)
Consumption of saliva fermented drinks		
No	7	(30,4 %)
Yes	16	(69,6 %)
History of viral hepatitis^a^		
No	13	(56,5 %)
Yes	10	(43,5 %)
HBV vaccination		
Incomplete	7	(30,4 %)
Complete	0	(0 %)
Unvaccinated	16	(69,6 %)

^a^Familiar or individual history of viral hepatitis

Anti-HDV antibodies (IgM or IgG) were detected in 43.5 % (10/23) of the HBsAg-positive samples. However none were positive for antibodies anti-HDV IgM and IgM. HBV DNA was detected in 9/23 samples and HDV RNA in 7/23 samples. One sample was positive for both HBV DNA and HDV RNA (Table 3).

**Table 3:** Serological and Molecular markers of HDV/HBV infection in Amerindians from Amazonas state, Colombia

Code of samples	IgM anti-HD	IgG anti-HD	Viral genome	HBsAg	anti-HBc total	IgM anti-HBc	Viral genome
608	+	-	+	+	+	-	-
992^a^	-	+	+	+	+	-	-
880	-	+	-	+	+	-	-
896	-	+	-	+	+	-	-
799	+	-	+	+	+	-	+
689	+	-	+	+	+	-	-
832	+	-	+	+	+	-	-
1137	-	+	-	+	+	-	-
1167	-	+	+	+	+	-	-
1141	-	+	+	+	+	-	-

^a^Symptomatic patient

**Fig. 1:**
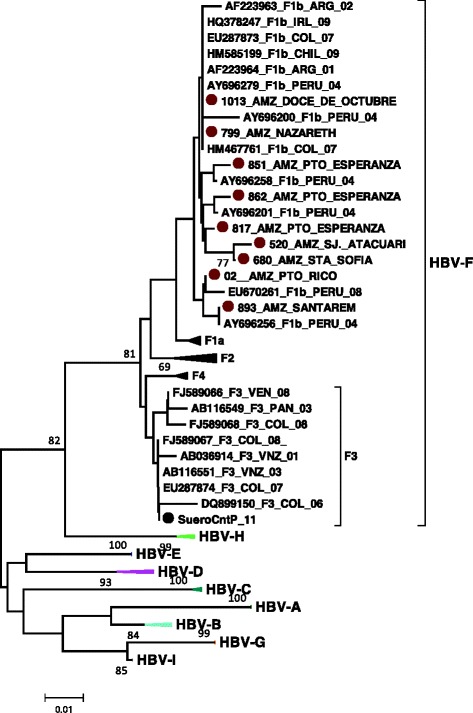
Unrooted phylogenetic tree of HBV ORF S generated by Neighbor Joining method. The sequences of this study (799, 1013, 851, 862, 817, 520, 680, 02 and893_AMZ) indicated with red circles, were compared with representative sequences of all HBV genotypes (A-I). The sequences are denoted with the GenBank accession number, genotype, country and year in which they were reported. The tree was generated using the software MEGA 5.1. Bootstrap values (indicated by arabic numbers) were obtained from 1000 replications. The black circle indicates the location of the positive control

Figure 1 shows the phylogenetic tree generated by the Neighbor Joining method for HBV. Isolates were grouped in the genotype F clade with bootstrap value of 81; all were classified as subgenotype F1b. Although the bootstrap values of the clade generated for genotype F1b is not strong, the samples (851_AMZ, 862_AMZ, 02_AMZ and 893_AMZ) were grouped in subgenotype F1b strains reported in Peru and Brazil [19].

**Fig. 2:**
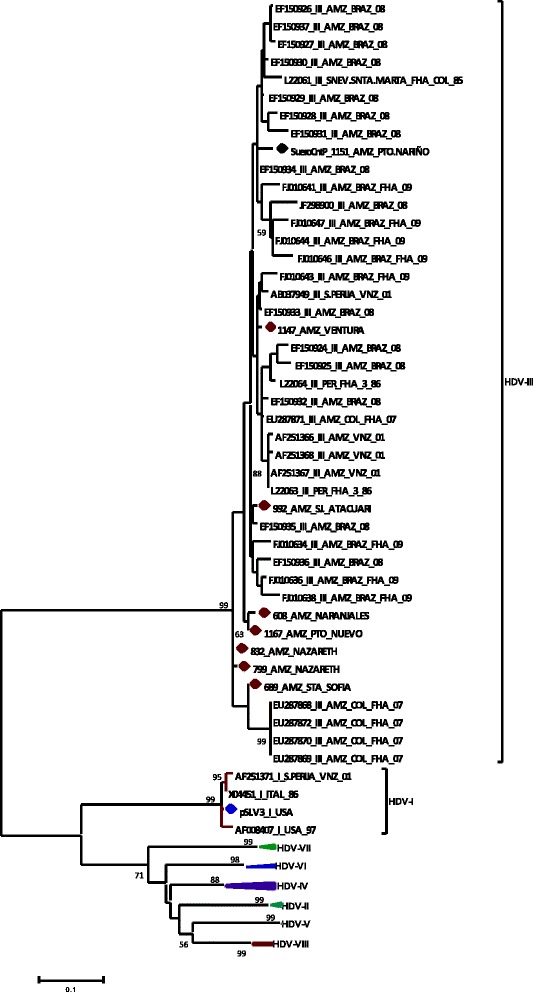
Unrooted phylogenetic tree of the ORF Delta of HDV, generated by maximum Likelihood method. Study sequences (1147, 992, 608, 1167, 832, 799 and 689_AMZ) indicated with red circles, were compared with sequences representative of all HDV genotypes [1–8]. The sequences are denoted with the GenBank accession number, genotype, country, acute liver failure diagnosis (FHA) and year in which they were reported. The tree was generated using the software MEGA 5.1. The bootstrap values (indicated by arabic numerals) were obtained from 1000 replication values. The black circle indicates the location of the positive control serum and the blue circle, the plasmid positive control

Regarding the HDV genotypes, the phylogenetic tree obtained by the Maximum Likelihood method showed that samples were grouped in the cluster of genotype 3 (bootstrap value of 99) (Fig. 2). The strains obtained from individuals of Amerindian communities as Nazareth (832_AMZ and 799_AMZ) and Santa Sofia (689_AMZ) were grouped into one clade with strains reported in 2007 from patients with acute liver failure in Amazonas state [18]; this grouping shows a possible nearest phylogenetic relationship between the strains. The strains isolated in Naranjales (608_AMZ) and Puerto Nuevo (1167_AMZ) communities were grouped in a single clade, suggesting a close phylogenetic relationship between these isolates. Additionally, the strain obtained in Ventura community (1147_AMZ) proved to be distant from other isolates in the study; this strain 1147_AMZ was grouped with strains from Amerindians in the Amazon basin of Brazil (EF150933 and FJ010643), Serrania Perija (AB037949) in Venezuela and Sierra Nevada de Santa Marta in Colombia (L22061) [3].

## Discussion

Colombia has a heterogeneous pattern for HBV infection. Although the average incidence registered in the period 2008–2014 to the Colombian health system (SIVIGILA) corresponds to low-intermediate endemicity (3.11, 3.64, 3.71, 4.2, 4,38 and 4.34/100.000), some states including Amazonas, Norte de Santander, Guaviare and Choco have been historically highly endemic for HBV infection [10–13,16,17].

Indeed, a very high prevalence of anti-HBc was reported in 1991 in children (0–9 years old 49 %, >10 years old 71 %) and women (61 %) population in Amazonas state [14]. These findings strongly suggested the implementation in 1992 of the HBV vaccination program, mainly in high-risk populations (children under 5 years). The effectiveness of this vaccination program was evaluated 8 years later and a substantially reduction of HBsAg prevalence (60–75 % reduction) was demonstrated in 2.145 Amerindian children under 12 years from Puerto Nariño and Leticia (Amazonas state) and Puerto Santander and Aracuara (Caqueta State) [20].

In the present study, 23 cases of HBV infection were identified from 862 Amerindians (861 asymptomatic and 1 symptomatic) from 19 communities of the rural area of Puerto Nariño, Leticia and Tarapaca in Amazonas state. A high frequency of HDV infection was observed in HBV-infected Ameridians (43.5 %, 10/23). The marker anti-HDV IgM was identified in 4 cases and anti-HD IgG in 6 cases. All cases of HDV infection were HBsAg and anti-HBc IgM positive, but negative for anti-HBc IgM (Tables 2 and 3). No data of HBeAg marker and liver function was available from these cases. Unfortunately, no samples remained for additional analysis then it was not possible to classify the clinical stage of these individuals. Except, the symptomatic case that was classified as coinfection HDV/HDV with descompensated liver chirrosis and fatal outcome.

Superinfection HDV/HBV is usually confirmed by detection of anti-HDV IgM in patients HBsAg positive and anti-HBc IgM negative. However, anti-HDV IgM Abs usually persist in patients with chronic infection. Indeed, the anti-HDV IgM is a marker of active HDV infection and a predictor of treatment response as described by Mederacke et al. in patients with chronic hepatitis. High anti-HDV IgM titers were described in patients with liver inflammatory disease while low IgG titers were related with inactive cirrhosis. Even, anti-HDV IgM persisted over many years in patients with progressive liver disease and after treatment [21].

Moreover, the findings of a study carried out in Pakistan described the profile of HDV co-infection in 169 patients from 480 cases of HBV infection with different clinical spectrum of disease. The AST level was normal in 43.2 % (73/169) of the patients and 75.7 % were negative for HBeAg. Asymptomatic carrier HBV stage was described in 26.6 % of the patients [22].

Considering the findings of these studies and the data of the present study, the individuals identified in these Amerindian communities could be cases of coinfection HDV/HBV.

HDV infection has been reported in other Amerindian populations in the Amazon basin with variable frequencies among the countries (Table 4). The overall anti-HDV rate was 13.4 % in 7 Amerindian communities in Brazil [23]. In Piaroas and Yanomami Amerindian communities in Venezuela, anti-HDV antibodies were reported in 6.4 % and 4.3 %, respectively [24]. Similarly, in a study carried out in population from Peruvian Amazon Basin, the prevalence of anti-HDV was 39 % [25]. A frequency of 8.25 % anti-HDV antibodies was reported in Amerindians from Amazonas state in Colombia [26].

**Table 4:** Studies of HDV/HBV infection frequency in Amerindian populations of Amazon basin

Reference	Country	Population	Anti-HBc+	HBsAg+	^a^Anti-HD+
Cabezas C. et al, 2006	Peru	37 Amerindian Communities n = 870	59.7 %	9.4 %	39 %
Braga W. et al, 2001	Brazil	7 Amerindian communities n = 688	54.5 %	9.7 %	13.4 %
Duarte M. et al, 2010	Venezuela	Piaroas n = 414	27.4 %	5.1 %	6.4 %
		Yanomami n = 231	58 %	14.3 %	4.3 %
Alvarado M. et al, 2011	Colombia	4 ethnic groups n = 176	54.5 %	7.9 %	8.2 %^b^

^a^% individuals HBsAg positive

^b^% individuals HBcand/or HBsAg positive

The frequency of HDV infection found in the present study population may be due to the bias selection of HBV infection cases by the rapid test of HBsAg detection. The rapid test is useful in areas like the Amazonas that are difficult to access; although the use of this technique probably generated a bottleneck excluding individuals HBV infected with HBsAg levels below the detection limit (3,5 IU HBsAg/mL) of the test [27]. Nevertheless, a high frequency of HDV infection in these communities is not excluded considering the epidemiology pattern of the HBV and HDV infection in these communities during the last 30 years [13, 14, 16, 17, 20].

In the present study, all cases were associated with HDV 3 (*n* = 7) and HBV subgenotype F1b (*n* = 9) (Figs. 1 and 2). This result is consistent with previous reports of these genotypes in Amerindian populations in the Amazon basin [3, 18, 28–31].

The non-monophyletic clustering obtained for HBV and for HDV strains of this study, as well as the proximity of the strains with those obtained in Peru and Brazil, suggest that these viruses are circulating in these populations, maybe through inter-ethnic interaction of Amazon basin communities and could be related to different sources of infection.

In South America, several studies demonstrated the predominance of HDV 3 in Amerindian population, associated mainly with HBV genotype F (subgenotypes F3 and F1b); although some cases of coinfection with HBV genotype A and genotype D were reported [3, 6, 28, 30]. In Venezuela and in Brazil, coinfection HDV 1 with HBV genotypes F and D has been described [31, 32]. Additionally, HDV 8 has been described in two cases in Brazil [33].

The analysis of the HBV DNA sequence coding for the S region of the envelope proteins demonstrated a phylogenetic relationship (862_AMZ, 851_AMZ, 02_AMZ, 893_AMZ) with subgenotype F1b isolated from individuals in Peru (Fig. 1). The phylogenetic distribution of these sequences indicates that HBV F1b circulates among Amerindian communities of the Amazon basin, regardless the country.

The interaction among the Amerindian communities of the Amazon basin is very dynamic including friendship, matrimonial alliances, rituals and festivals, and political organizations [34]. Actually, the Tikuna is the main community in Peru and in South East Colombia. Such ties could explain the movement of these strains between the countries and the prevalence of HBV subgenotype F1b in these populations.

However, studies in other populations in Colombia (blood donor population and patients with end-stage liver disease) showed the prevalence of HBV subgenotype F3 [35, 36]; however, none of the samples of the present study correspond to subgenotype F3.

A recent study performed in two municipalities of Colombia, Quibdo (Pacific Region) and Apartado (North east region) characterized the HBV genotypes in asymptomatic people with risk factors for HBV infection. The genotype F was found in both population (subgenotype F3 and F1a) and genotype A was only described in Quibdo. It should be noted that the population predominant in Quibdo are Afro-descendant, while in Apartado there are different ethnic groups, including Caucasian, Mestizo and Afro-descendants [37]. This result is in agreement with the report of Alvarado et al. in Afro-descendant individuals from Quibdo with a predominance of HBV subgenotype A [38].

The findings of these studies suggest that HBV genotype F3 is the most frequent in populations from the Andean region of Colombia (Antioquia, Bogotá and Bucaramanga), HBV genotype A in the Pacific region (Quibdo) while HBV subgenotype F1b could be the predominant in South East (Amazonas state) [18, 35–38].

The circulation of different HBV genotypes could be related to the numerous ethnic groups present in Colombia. Indeed, the estimated admixture population in Colombia is American ancestry 0.29, European ancestry 0.6 and African ancestry 0.1; although African ancestry is highest in the Pacific and Atlantic regions (particularly on the Pacific) and the highest European ancestry is in the central region of Colombia. Meanwhile, American ancestry is highest in the south of the country [39].

Several limitations of this study need to be considered which a rapid test was used for selection of the individuals HBsAg positive. Importantly, the epidemiological data was obtained only from the 23 individuals HBsAg positive as well the blood samples. Moreover, the serum sample was not available for liver function marker. Finally, a follow-up of the patients should be performed to determine the clinical stage and the treatment.

In conclusion, coinfection HBV/HDV was frequent in the study population (43.5 %, 10/23). Nine cases were identified in a population of 861 asymptomatic Amerindian individuals; one symptomatic case (with diagnosis of end-stage hepatic disease) was also identified in the study. The circulation of HDV 3 and HBV subgenotype F1b in this population suggests a constant flow of these viral genotypes in Colombia, as a result of the intra and inter-ethnic interaction of the Amerindian population from Amazon basin.

The findings of this study are important for public health authorities in Colombia considering the important number of HDV/HBV cases identified in asymptomatic population in Amazonas state to establish a protocol for diagnosis and management of viral hepatitis in Amerindian population.

Further studies are necessary to confirm whether HBV subgenotype F1b is the prevalent in Amerindians from South East region in Colombia (Amazonas, Caqueta, Vaupes, Guaviare and Putumayo states) and whether other HBV genotypes have been recently introduced. Additionally, it would be very interesting to evaluate the HBV evolution in these Amerindian communities.

## Methods

### Study Area

**Fig. 3:**
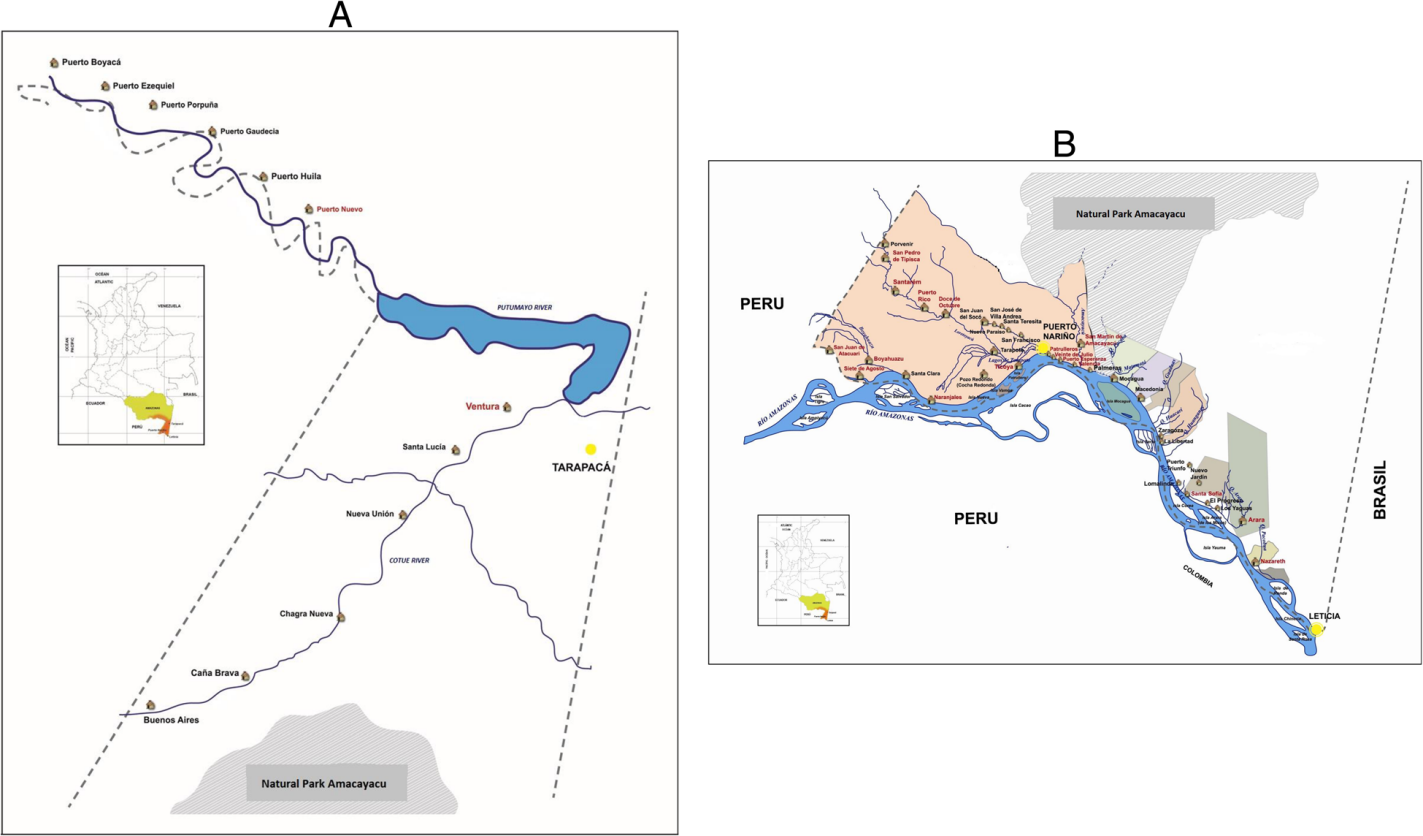
**a.** Map of North in Amazonas State in Colombia. Geographical location of the Amerindian communities in rural área of Tarapaca village included in the study. **b.** Map of South in Amazonas State in Colombia. Geographical location of Amerindian communities in rural areas of Puerto Nariño and Leticia municipalities included in the study

The study area is located in the Amazonas state, South East Colombia. This state has approximately 74.000 inhabitants mainly Amerindians (72 %) that belong to different ethnic groups including Tikuna, Cocama, Yagua, Huitotos and Muinanes [34]. The study was carried out in 19 Amerindian communities of the rural zone of Tarapaca Village and Puerto Nariño and Leticia municipalities. These communities are settled on the banks of the Putumayo River and a tributary (Cotue) (Fig. 3a) and the Amazon River and its tributaries (Boyahuazu, Amacayacu and Loretoyaku rivers) (Fig. 3b).

### Study population

Amerindian men and women, over 18 years of age, who voluntarily agreed to participate by signing the informed consent, were recruited for the study between June 2011 and May 2013. The population was invited to participate in the study through home visits and meetings in each one of the communities.

All procedures adopted in this study were followed the terms established by the Ethic Committee of Universidad Nacional (N.14, Faculty of Medicine, Universidad Nacional). The project was approved by the health authorities of the Amazonas state and by the Amerindian communities.

### Serological and molecular markers of HBV infection

Rapid tests for the detection of HBsAg (HBsAg determine Orgenic Kit; Alere, USA; detection limit 3,5 IU HBsAg/mL) were carried out on the study population. Individuals positive by the rapid test were interviewed by one of the researchers, who completed a demographic questionnaire (age, gender, birthplace, Amerindian community, municipality, ethnicity, social security and education) and epidemiological variables (history of jaundice, history of hepatitis, risk factors of viral hepatitis, Hepatitis B vaccination status). After that, blood samples were collected from these individuals. Serum samples were stored at −4 °C until the time of transportation to the laboratory. The samples were kept at −70 °C.

All serum samples were analyzed using commercial ELISA kits for detection of HBsAg (ABBOTT PRISM HBsAg assay, USA) and total anti-HBc (ABBOTT HBcore PRISM assay, USA) following the manufacturer’s instructions. The samples were analyzed in duplicate.

DNA was extracted from the serum using a commercial kit (Qiagen, The Netherlands). The HBV genome was detected by nested polymerase chain reaction (nested-PCR) of ORF S (nt 422–758). Amplification conditions and cycling were modified from protocols previously published [40, 41]. Primers used in the first round were YS1 and YS2 [39]. The PCR conditions were 3 min 93 °C, 35 cycles 45 s 94 °C, 1 min 53 °C and 1 min 72 °C, and 6 min 72 °C. Nested-PCR was performed using the primers S3s and S3as [38]. The cycling conditions were 4 min 94 °C, 40 cycles 1 min 95 °C, 1 min 50 °C and 1 min 72 °C, and 7 min 72 °C.

### Serological and molecular markers of HDV infection

HBsAg-positive serum samples were analyzed for anti-HD IgM and IgG using commercial ELISA kit (HDV IgM and IgG; DIA.PRO, Italy) following the manufacturer’s instructions. All samples were tested in duplicate. Serum samples that were HDV positive for molecular/serological markers were tested for anti-HBc IgM (Abbott anti-HBc-IgM test; USA).

Viral RNA extraction was performed using a commercial kit (QIAamp Viral RNA Mini Kit, Qiagen; Germany). Detection of the HDV genome was performed by RT-nested PCR (delta ORF nt 877–1290). Amplification conditions and cycling were modified from protocols previously published [3, 42]. Briefly, reverse transcription was performed at 37 °C for 60 min (MMLV, Invitrogen, USA), using the primer 1302D. Primers and conditions of the first round PCR were 8531U and 1302D, 2 min 94 °C, 40 cycles 30 s 94 °C, 30 s 56 °C and 1 min 72 °C and 10 min 72 °C. For the second round PCR, the primers HDV-E and HDV-A were used. The cycling conditions consisted of 4 min 94 °C, 40 cycles 1 min 95 °C, 1 min 50 °C and 1 min 72 °C, and 7 min 72 °C.

### Viral genotype characterization

Nucleotide sequences of amplicons were determined by automated dideoxysequencing (Macrogen, Inc. Seoul, Korea). Sequences were aligned with Clustal X and edited using Bioedit 7.1.11. Unrooted neighbor-joining and maximum-likelihood trees were constructed using the Mega 5.1 (Molecular Evolutionary Genetics Analysis) software. The best model of nucleotide substitution was *Kimura-2-parameter + Gamma distribution* for HBV, and model of nucleotide substitution model for HDV corresponded to *Hasegawa-Kishino-Yano + + Invariably Gamma distribution.* Reliability of the trees was evaluated statistically by bootstrap analyses with 1000 replicates. Bootstrap values higher than 70 % were considered significant. Genotypes were determined according to the clustering of 49 HBV and 56 HDV sequences available from the GenBank database (National Center for Biotechnology Information, National Institutes of Health).

### Statistical analysis

For quantitative variable the mean, median and standard deviations were obtained. The information was tabulated in MS Excel 2010. For statistical analyzes, the SPSS (Statistical Product and Service Solutions) software version 14.0 was used.

## Abbreviations

HDV: Hepatitis D virus
HBV: Hepatitis B virus
SIVIGILA: Surveillance system of public health in Colombia
HBsAg: Hepatitis B surface Antigen
anti-HBc: Antibodies anti HBV Core protein
ORF: Open reading frame
SDAg: Small delta antigen
LDAg: Large delta antigen
anti-HDV: Antibodies anti HDV
IgG: Immunoglobulin G
IgM: Immunoglobulin M
PCR: Polymerase chain reaction
RT: Retro-transcription
MMLV: Moloney murine leukemia virus
Mega 5.1: Molecular Evolutionary Genetics Analysis
ACITAM: Asociacion de Cabildos Indigenas del Trapecio Amazonico
ATICOYA: AsociacionTikuna, Cocama y Yagua
CIMTAR: Cabildo Indigena Mayor de Tarapaca
SPSS: Statistical Product and Service Solutions
WHO: World Health Organization
IU: International Units
FHA: Acute liver failure diagnosis
